# Short-Term Therapeutic Effect of Repetitive Transcranial Magnetic Stimulations of Sleep Disorders in Parkinson’s Disease: A Randomized Clinical Trial (Pilot Study)

**DOI:** 10.3390/brainsci14060556

**Published:** 2024-05-30

**Authors:** Eman M. Khedr, Gellan K. Ahmed, Mohammad Ahmad Korayem, Sara Ahmed Salah Hussain Elamary, Maha M. El-kholy, Nourelhoda A. Haridy

**Affiliations:** 1 Department of Neurology and Psychiatry, Faculty of Medicine, Assiut University, Assiut 71515, Egypt; gillankaram@aun.edu.eg (G.K.A.); mohamed.20134267@med.aun.edu.eg (M.A.K.); nourelhodaahmed@aun.edu.eg (N.A.H.); 2 Assiut University Hospitals, Faculty of Medicine, Assiut University, Assiut 71515, Egypt; sara.16285213@med.aun.edu.eg; 3 Department of Chest Diseases and Tuberculosis, Faculty of Medicine, Assiut University, Assiut 71515, Egypt; maha_elkholy@yahoo.com

**Keywords:** repetitive transcranial magnetic stimulation, sleep quality, insomnia, polysomnography, Parkinson’s disease

## Abstract

This study aimed to evaluate the efficacy of rTMS in treating sleep disorders in PD. It included 24 patients with PD who had sleep disorders. Group allocations (active or sham with a ratio of 2:1) were placed in serially numbered closed envelopes. Each patient was evaluated with the following: MDS-UPDRS, Parkinson’s Disease Sleep Scale (PDSS), Beck Depression Inventory (BDI), and polysomnography (PSG) before and 10 days after the treatment sessions. Each session consisted of 10 trains, 20 Hz, 10 sec for each, over the parietal cortex (bilaterally). Scores of UPDRS, BDI, and PDSS improved significantly in the active group but not in the sham group. The PSG data showed that sleep onset and rapid eye movement (REM) latencies (min), REM duration, and time spent awake (both as %TST) were improved after rTMS in the active group compared with the sham group. The number of awakenings, the wake-after-sleep onset index, the arousal index, and periodic leg movements (PLMs) were all significantly reduced in the active group but not in the sham group. Ten sessions of 20 Hz rTMS over parietal cortexes improved sleep quality and PLMs in patients with PD. The improvement in PSG and PDSS were correlated with improvements in UPDRS and BDI scores.

## 1. Introduction

The average prevalence rate of Parkinson’s disease (PD) in Upper Egypt governorates is among the highest in the world, ranging from 557 to 436 cases per 100,000 people in Assiut and Qena governorates, respectively [1,2]. Overall, 60 to 98% of patients with PD report disrupted sleep, making it one of the most common non-motor symptoms and contributing substantially to a diminished quality of life [3]. Previous research found that 78.6% of 112 patients diagnosed with PD experienced sleep disturbances. The most commonly reported complaint was difficulty initiating or maintaining sleep at night (46.4%), followed by vivid nightmares and night terrors (27.7%) [4]. Other studies observed that 80–90% of patients with PD suffer from sleep disturbances [5] and nearly all patients develop such symptoms at some point during the course of the disease [6]. The pathophysiology underlying sleep disturbances in PD remains unclear. One primary factor is the degeneration of major sleep regulatory centers in the brainstem [7]. In addition to dopaminergic medications, sleep disturbances may also be exacerbated by nocturnal akinesia, depression, or restless legs syndrome [8]. Sleep disturbances were previously associated with dopaminergic dysfunction in the hypothalamus [9]. In addition, PD is associated with disruptions in functional connectivity within and between brain networks involved in sleep regulation [10,11].

Repetitive transcranial magnetic stimulation (rTMS) is a non-invasive technique that activates the cerebral cortex [9] and has been employed as a non-pharmacological therapy for treating motor symptoms in PD, albeit with varying degrees of success [12]. In healthy individuals, rTMS was shown to affect sleep organization both before and during sleep [13].

Huang et al. 2018 investigated how low-frequency rTMS over the parietal cortex impacted the Pittsburgh Sleep Quality Index (PSQI) and Hamilton Rating Scale for Anxiety (HRS-A) scores in individuals with comorbid insomnia and generalized anxiety disorder. Their findings indicated that active rTMS improved PSQI scores more than sham stimulation, making this the second sham-controlled experiment to demonstrate a significant effect without a placebo response [14]. Furthermore, a significant association was observed between the improvement in HRS-A scores and PSQI scores, suggesting that enhanced sleep quality may be linked to reduced anxiety levels. However, it remained unclear whether rTMS had an intrinsic role in sleep regulation or if the reductions in anxiety and insomnia were independent effects [15].

In another study, Jiang et al. (2013) evaluated the efficacy of rTMS, pharmacotherapy, and cognitive behavioral therapy in treating chronic insomnia. They employed PSQI and polysomnography (PSG) for assessments. According to the PSG data, rTMS was the only treatment modality that enhanced slow-wave sleep (stage 3) and rapid eye movement (REM) sleep [16]. rTMS has been applied over several different anatomical locations to treat insomnia such as the DLPFC bilaterally, right parietal cortex, and dominant primary motor cortex (M1); treatment of RLS has targeted the M1 leg area bilaterally, left primary somatosensory cortex, and left M1 [17].

Despite the number of studies on the impact of rTMS in primary insomnia, there are few studies using rTMS to treat sleep disorders in PD, particularly using objective measures of sleep symptoms and structure. Two studies using rTMS for sleep disorders in PD [18,19] applied high-frequency (HF) rTMS over the motor and parietal cortexes, respectively, and reported results with subjective and objective measures. In one of them, polysomnographic (PSG) recordings showed a reduction in the frequency of arousal from sleep; in the other, actigraphy recordings in non-REM stage I improved [18]. One sham-controlled trial investigating the effects of rTMS on sleep in individuals with PD observed significant placebo-related improvements in subjective questionnaire measures (PDSS, HDRS, UPDRS) as well as equal improvement in sleep [20]. Recently, sleep quality and excessive daytime sleepiness in patients with PD were also reported to improve after rTMS [21]. Recently, Shaheen et al. (2023) explored the effect of HF rTMS over the parietal cortex on sleep quality in patients with Parkinson’s disease (PD). They found subjective improvement in sleep disorders [22].

As the parietal cortex is involved in the regulation of arousal and sleep–wake cycles through its connections with the thalamus, applying rTMS over the parietal cortex may modulate the activity of thalamocortical networks. This may influence the regulation of sleep–wake cycles, potentially improve sleep quality [23,24], and help to restore or modulate functional connections, potentially improving the communication and coordination among brain regions involved in sleep processes [10,11].

Given the mixed results in the literature, the present study aimed to evaluate if HF rTMS over the parietal cortex has a direct impact on the sleep quality of PD patients using both objective and subjective scores and to test whether this correlates with changes in depression and motor performance.

## 2. Materials and Methods

Forty-five patients with PD were recruited from inpatient and outpatient clinics of the neuropsychiatric department of Assiut University Hospital. All patients fulfilled the diagnostic criteria for Parkinson’s disease as outlined by the U.K. Brain Bank [20]. Of the total cohort, 30 patients reported experiencing sleep disturbances based on their responses to the self-reported Non-Motor Symptoms Questionnaire (NMSQuest) (the specific questions related to sleep disturbance were 23, 24, 25, and 26) [25].

The exclusion criteria included individuals with PD who have impaired consciousness, significant cognitive decline with moderate to severe decreases in MMSE scores, psychosis, medical conditions such as renal failure, liver cell failure, respiratory failure, endocrine dysfunction, and other forms of Parkinsonism such as multisystem atrophy, supranuclear palsy, and encephalitic Parkinsonism. Patients were also excluded if they had any contraindications for magnetic stimulation (metallic objects in the skull, pacemaker, epilepsy). Treatment was maintained constant during the trial. All participating patients were asked not to use hypnotic drugs or receive other treatments related to insomnia two weeks before starting sessions. All rTMS sessions were performed during the daytime while the participants were awake. Each patient received dopaminergic and anticholinergic drugs with carbidopa/levodopa (Sinemet) 250/25, half a tablet three or four times daily, amantadine hydrochloride (NMDA receptor inhibitor) twice daily, and anticholinergic (Cogentin) twice daily.

### 2.1. Sample Size Calculation

The sample size was determined using G*Power3.1 software based on previous study [26] with an alpha level of 0.05, a power of 0.80, and an allocation ratio (N2/N1) of 1. A two-tailed test was employed for data analysis.

### 2.2. Methodology

#### 2.2.1. Clinical Assessment

Each patient was assessed with the following: Parkinson’s Disease Sleep Scale (PDSS) [27], cognitive function using the Arabic version of the Mini-mental State Examination (MMSE) [28], Arabic version of Movement Disorder Society-Sponsored Unified Parkinson’s Disease Rating Scale (MDS-UPDRS) [29], which had good construct validation by confirmatory factor analysis (CFA) (CFA ≥ 0.90), Beck Depression Inventory-II (BDI-II) [30], and polysomnography.

##### Beck Depression Inventory-II (BDI-II) [30]

The BDI-II comprises approximately 21 items, with each response being evaluated on a scale of 0 to 3. Depressive symptoms that are more severe are indicated by higher total scores [31]. It demonstrated acceptable reliability and validity, with a range of 0.73 to 0.92 [32].

#### 2.2.2. Polysomnography

All participants (active and sham) underwent full-night attended polysomnography (Somnstar 4100, Sensor-Medicus Co, Yorba Linda, CA, USA) in the sleep lab of the chest department, Assiut University Hospital. This was performed twice, before and after the end of rTMS treatment sessions. The following physiological assessments were documented: electromyogram of the chin (EMG), electrocardiogram (EKG), electrooculogram (EOG), electroencephalogram (EEG) (C3-A2, C4-A1), body position, limb movements, pulse oximetry, nasal and oral airflow, thoracic and abdominal effort, and snoring sound level.

The resting motor threshold (RMT) was measured with a figure-8 coil connected to a Magstim model 200 stimulator (Magstim, Whitland, UK), which had a maximal output of 2.2 T. We located the optimal scalp hot spot for the hand area of each hemisphere as the location from which motor-evoked potentials (MEPs) of greatest amplitude could be recorded from first dorsal interosseous (FDI) muscle [33]. A Nihon Kohden Machine model 9400 (Tokyo, Japan) was used to amplify and record the signals.

### 2.3. Randomization (Parallel Design)

**Figure 1:**
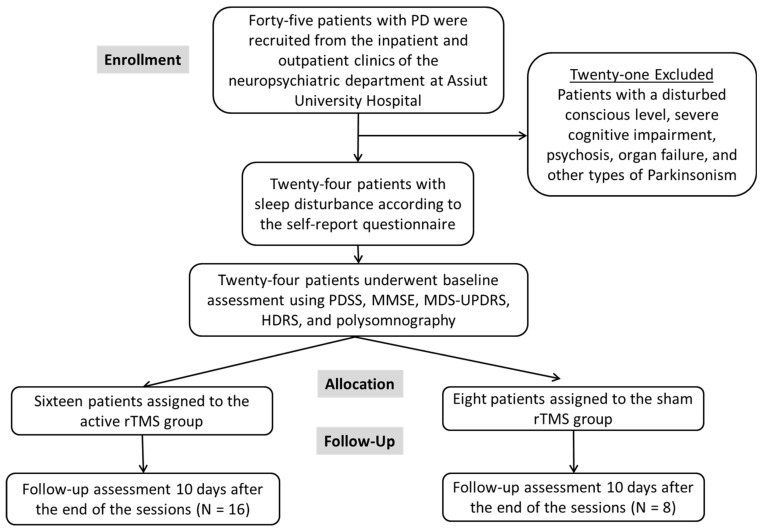
Flowchart showing the number of patients with Parkinson’s enrolled, excluded, allocated, and followed after rTMS.

Out of the 45 patients with PD, 30 had sleep disturbance. Out of them, 6 individuals declined to participate, resulting in a total of 24 participants in this study (see Figure 1).

Group allocations: The sequentially numbered, opaque, sealed envelope technique was employed to randomly allocate patients with PD and sleep disorders to one of two treatment groups. The real and sham groups were divided into serially numbered, opaque closed envelopes at a 2:1 ratio. Once a patient consented to participate, the investigator opened the sealed envelope and subsequently designated the treatment group by opening the corresponding envelope. This study was double-blind (participant and the outcomes assessor): the participants were not notified of the potential alternative therapies used in the trial, and the outcome assessor was unaware of the treatment that the participants underwent.

### 2.4. Repetitive Transcranial Magnetic Stimulation Procedure

Initially, we evaluated the resting motor threshold. Patients in the active group underwent repetitive transcranial magnetic stimulation (rTMS) targeting the right then left parietal areas (P4 and P3, according to the 10–20 system). This treatment consisted of ten sessions, with each session consisting of 10 trains of stimulation at a frequency of 20 Hz. Each train lasted for 10 s, with 30 s as the inter-train interval. The stimulation intensity was adjusted to 80% of RMT of the first dorsal interosseous (FDI) muscle in the contralateral hand with a total of 2000 pulses for each parietal area. The sham group underwent rTMS with similar parameters but with the coil edge positioned perpendicular to the scalp in the sagittal plane to mimic the stimulation noise. Each patient underwent a total of ten sessions, receiving five sessions each week for two consecutive weeks. The participants were mandated to attend all sessions during this study and were not permitted to change their treatment plan.

### 2.5. Follow Up

Every patient underwent follow-up ten days after the termination of treatment and was assessed using the Beck Depression Inventory, PDSS, MDS-UPDRS parts II and III (motor sections), and polysomnography.

Ethical approval and written informed consent:

This research received approval from the academic and ethical committee of Assiut University. Informed written consent was obtained from each patient prior to undergoing the operation. The protocol was registered at clinicaltrial.gov with ID: NCT05599035.

### 2.6. Statistical Analysis

The statistical analysis was conducted using SPSS (version 26). Before starting the stimulation regimen (at PRE), each variable was checked using the Shapiro–Wilk Test to test the normality of data. Qualitative data were analyzed using frequency and percentage analysis, while quantitative values were given as mean ± standard deviation (SD), median, and range. As most of the variables were not normally distributed, the Pearson chi-square test was employed to compare categorical variables, the Mann–Whitney U test was utilized to compare mean values between two independent groups, and the Wilcoxon signed rank test was employed to compare before and after measures within the same group. The Spearman correlation coefficient is used to measure the relationship between variables. In addition, Friedman analyses were conducted. To assess the number of patients who developed improvement after treatment sessions, the % of improvement was measured as follows: Score of each scale pre-post 10 session/pre score × 100. An arbitrary 30% or more was considered an improvement. To assess the magnitude of differences between the two groups, we employed Cohen’s d as a measure of effect size. This metric provides insight into the practical significance of the observed differences, going beyond mere statistical significance. A *p*-value of 0.05 was deemed to have statistical significance.

## 3. Results

The baseline data for the studied groups are listed in Table 1. There was no significant difference among the groups in demographic and clinical rating scales.

The effect of rTMS on the different rating scales is listed in Table 2. There were significant changes in the rating scales following active treatment, while no such changes were observed in the sham group. This led to a significant group X time interaction in many of the scores. In addition to statistical significance, the clinical relevance of our findings was assessed using effect size measures. For instance, the effect size for the improvement in PDSS in the active group was large (Cohen’s d = 3.724), indicating a substantial clinical improvement. Similarly, improvements in MDS-UPDRS and BDI-II also showed large effect sizes (Cohen’s d = 3.71 and 3.269, respectively), supporting the clinical significance of rTMS treatment in improving motor function and depressive symptoms in patients with Parkinson’s disease.

**Figure 2:**
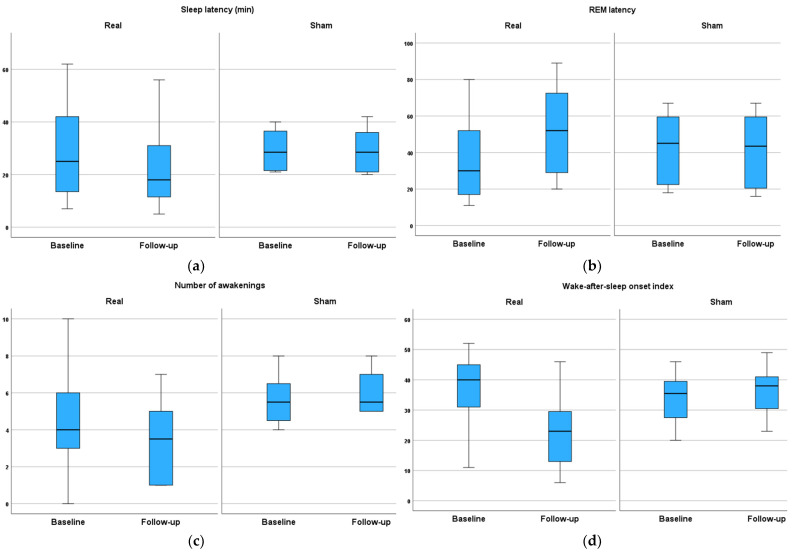

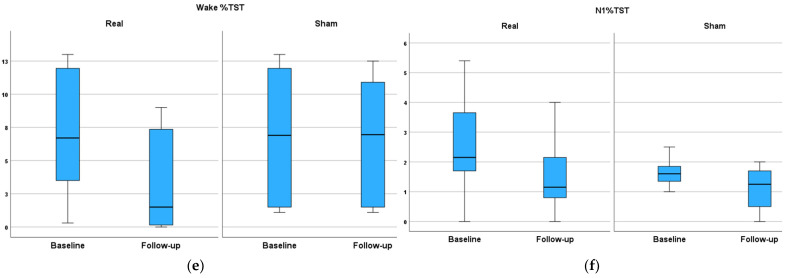
The effect of rTMS on different parameters of polysomnography (pre- and post-10 treatment sessions. The mean values of the parameters of PSG (pre- and post-10 treatment sessions) of (**a**) sleep latency, (**b**) REM latency, (**c**) number of awakenings, (**d**) wake-after-sleep onset index, (**e**) wake %TST, and (**f**) N1%TST among studied groups.

The effect of rTMS treatment sessions on polysomnography (PSG) parameters (part 1) is listed in Table 3 and shown in Figure 2. The PSG data at baseline before treatment sessions showed that both groups had a prolonged sleep latency and reduced REM latency compared with normal data from our lab. A long sleep latency means that it can take more than 20 min to fall asleep at night. A long sleep latency can be a sign of insomnia. A REM latency that is shorter or longer than usual may be caused by sleep deprivation or sleep disorders. They also had an increase in the number of awakenings and the wake-after-sleep onset index, meaning sleep fragmentation. In addition, stage N1 sleep is an estimate of the degree of sleep fragmentation. The high percentage of stage N1 sleep recorded in both groups is generally a result of frequent arousals caused by sleep disorders.

After active rTMS treatment, sleep latency and REM latency (min) both improved, while there were no such changes in the sham group. There was a significant group × time interaction for both measures (*p* = 0.003, and 0.007, respectively).

The number of awakenings decreased, and the wake-after-sleep onset index improved significantly in the active, but not the sham, group (*p* = 0.007 for each parameter). No changes in sleep efficiency were recorded in either group.

Wake % TST and N1 % TST decreased significantly after active treatment with a significant group × time interaction (*p* < 0.001 for both). REM% TST also increased (improved) in the active group. There were no changes in N2 and N3 % TST (deep sleep) in either group.

The effect of rTMS treatment sessions on polysomnography parameters (part II) is listed in Table 4. At the baseline assessment, there was a mild degree of desaturation index (ODI) with median values of 5.7 in the sham and 5.9 in the real group (normal ODI < 5 events/hour). There was no change after treatment in either group.

Prior to treatment, there were mild but not significant increases in the mean and median values of the arousal and PLM indices (mild: 5–14.9 events/hour) in both groups compared with the normal data from our lab. However, there was a significant reduction (improvement) in both scores after active rTMS, while no such changes were observed in the sham group (time × group interaction *p* = 0.033 and 0.001 for the arousal index and PLM, respectively).

There was a significant improvement in the hypopnea index AHI/h after real rTMS (*p* = 0.019) but not in the sham group, although there was no significant time × group interaction.

The correlations between changes in the rating scales and the PSG results are listed in Table 5. The mean changes in PDSS were significantly correlated with sleep latency and negatively correlated with REM latency (*p* = 0.022 and <0.0001, respectively). Changes in UPDRS parts II and III were significantly correlated with sleep latency and N1 % TST as well as negatively correlated with REM latencies and REM % TST (*p* = 0.004). The mean changes in the BDI scale were significantly correlated with REM % TST (*p* = 0.004) and negatively correlated with the wake-after-sleep onset index (0.04).

## 4. Discussion

A wide range of sleep disturbances may occur as comorbidities in patients with PD, which has an impact on individuals’ physical and mental health. The most common sleep disorders in PD are difficulty falling asleep, disturbances occurring during sleep, abnormal movements during sleep, insufficient sleep, and excessive sleep [34]. This study aimed to evaluate the therapeutic effect of HF rTMS over the parietal cortex (bilaterally) on sleep disturbance in patients with PD. The main findings of the present study were that compared with the sham, active rTMS had (1) a significantly greater beneficial effect on all clinical rating scales (PDSS, MDS-UPDRS motor sections II and III, and BDI) pre- and post-sessions and (2) produced a greater objective improvement in sleep patterns, as confirmed by PSG. Finally, improvements in PDSS and PSG were partially correlated with changes in both UPDRS and BDI.

Many factors contribute to poor sleep quality in PD, such as motor symptoms of bradykinesia, rigidity, motor fluctuations, and PLM [35,36] as well as neurodegeneration in parts of the brainstem (basal ganglia and substantia nigra) and hypothalamus that control sleep (hypothalamic volume loss is associated with reduced melatonin output in PD) [37]. PD is associated with disruptions in functional connectivity within and between brain networks involved in sleep regulation [10,11]. There are also direct effects of dopaminergic medications on waking people up [36].

In the present study, the improvement in PDSS after HF rTMS over the parietal cortex can be explained by the area’s recognized involvement in sleep-regulating processes. Indeed, the results were consistent with previous studies in both primary insomnia [38] and sleep disturbance in PD [18,19,22]. Consistent with our stimulation of the parietal cortex, Shaheen et al. (2023) [22] explored the effect of HF rTMS over the parietal cortex on sleep quality in patients with PD using subjective scales and found the same result. The effect of HF rTMS on both motor performance using UPDRS-III and sleep disorders using the self-assessment scale in PD through stimulation of the primary motor cortex (PMC) was investigated previously by our group in a randomized clinical trial [39]. We found more improvement in both scales than a placebo effect. Van Dijk et al. [18] found an objective improvement in patients’ sleep patterns (sleep fragmentation and sleep efficiency) and reduced duration of nocturnal awakenings after 10 days of 5 Hz rTMS over the motor or parietal cortex using actigraphy, which is a diagnostic tool [18]. The enhancement in sleep quality following the stimulation of the parietal cortex can be attributed to the well-documented role of this brain region in sleep-regulating processes [40]. The stimulation of PMC may improve sleep secondary to the improvement in motor performance. Antczak et al. found that HF (15 Hz) rTMS for 10 days over both PMCs improved subjective and objective sleep quality, as reflected by a decreased frequency in arousal from sleep and in Non-REM–1 stage sleep [19]. Arias et al. (2010) reported similar improvements in PDSS after HF rTMS at the vertex, although the same effect occurred in a sham group. Interestingly, there was no change in actigraphy scores in the sham group even though the PDSS improved. This underlines the fact that a placebo response causing subjective improvements did not show up objectively in the “sleeping” patient [20].

The improvement in PDSS following active rTMS was also associated with improvements in MDS-UPDRS II and III. Our results are consistent with our previous studies [39,41,42], which found that HF-rTMS improved motor disability in patients with PD when applied over the primary motor area (M1) of both hemispheres. Given the connections between the parietal and motor cortexes [43], this could perhaps account for some of the effects on the UPDSR. Alternatively, improving sleep quality itself could drive improvement in motor scores [14,44,45].

Sleep disorders and insomnia frequently go hand-in-hand with anxiety and depression [46]. Depression may be a contributing factor to sleep problems in people with PD. In the present study, active HF rTMS over the parietal cortex had a beneficial effect on the BDI scale, which was significantly correlated with the improvement in PDSS. Previous studies have shown that HF rTMS can improve depressive symptoms in PD and improve sleep quality, although the target region for rTMS was the frontal cortex rather than the parietal cortex [47,48]. The present findings might therefore be explained by a reciprocal effect, meaning that an improvement in sleep quality by rTMS of the parietal cortex leads to a secondary improvement in depression.

Our results confirm those of van Dijk et al. (2009), who found that stimulation of the parietal cortex improved patients’ sleep profiles [18]. Nevertheless, that investigation documented sleep via actigraphy, a diagnostic modality considered to be less precise than nocturnal polysomnography (PSG), specifically in terms of ascertaining the proportion of distinct sleep phases, the existence of electroencephalogram (EEG) arousals, and sleep-related respiratory disorders.

The present detailed results show that there was a significant decrease in both sleep latency and the wake-after-onset of sleep, indicating an improvement in insomnia. Also, there was a reduction in the number of awakenings and a decrease in the wake-after-sleep onset index. The wake % and light sleep (N1) % TST were significantly decreased in the active group, meaning decreased fragmentation and restored normal sleep.

In the present study, the % stage of light sleep (N1 %) and the arousal index both were decreased, which is consistent with Antczak et al. (2011), who reported that rTMS reduced episodes of light sleep (N1%) and the arousal index (AI) in 11 patients with PD who had ten daily 15 Hz rTMS sessions [19]. The drop in N1 % among our patients is due to the decreased AI and supports the conclusion that patients with PD have more arousals during light sleep. The percentage of N1 sleep has also decreased in a study by Graf et al. [13], although this was administered over the left dorsolateral prefrontal cortex rather than the parietal cortex.

N2 and N3 % TST (deep sleep) showed no significant changes after treatment stimulation in either group. Interestingly active rTMS produced a substantial decrease in the PLM index with no change in respiratory events, and the apnea–hypopnea index decreased. Again, these are consistent with Antczak et al., who found that PLM and sleep-related respiratory issues remained unchanged [19].

Finally, the correlation between changes in different rating scales (PDSS, MDS-UPDRS motor section, and BDI scales) with the parameters of the PSG suggests that the improvement in sleep quality caused by rTMS is intimately related to the associated improvement in motor and depressive symptoms.

There are several possible potential mechanisms by which HF rTMS over the parietal cortex could improve sleep quality in patients with PD and sleep disorders. Firstly, the parietal cortex, particularly the primary somatosensory cortex (S1), is believed to be hypofunctional in patients with PD and sleep disturbances, and high-frequency rTMS may partially reverse this parietal hypofunctionality [18]. Secondly, PD is associated with disruptions in functional connectivity within and between brain networks involved in sleep regulation. HF rTMS applied over the parietal cortex may help to restore or modulate these functional connections, potentially improving the communication and coordination among brain regions involved in sleep processes [10,11]. Thirdly, the parietal lobe and hippocampus are known to be involved in sleep processes, and there is evidence suggesting that parietal rTMS may indirectly affect hippocampal function through their anatomical and functional connections. Enhancing the functional connectivity between the parietal cortex and hippocampus by HF rTMS may regulate sleep-related processes [49]. Additionally, rTMS may trigger the release of neurotransmitters crucial for the sleep–wake cycle, such as dopamine, melatonin, serotonin, noradrenaline, and GABA, contributing to better sleep quality [50]. Moreover, rTMS over the parietal cortex may improve concomitant motor disabilities and depressive symptoms in patients with PD, as documented in the present study, which could indirectly enhance sleep quality by reducing the disruptive effects of these symptoms on sleep.

The main strength of this study was the inclusion of subjective and objective measures to evaluate the effect of rTMS on sleep.

One of the limitations of this study is the small sample size, which may increase the risk of type 2 error, potentially leading to non-significant findings despite actual effects. Future studies with larger sample sizes are necessary to confirm these results and further investigate the long-term effects of rTMS on sleep disturbances in patients with Parkinson’s disease. Additionally, while we reported effect sizes to convey the clinical significance of our findings, these should be interpreted with caution given the pilot nature of this study. Another limitation of the present study is the sleep assessments that occurred immediately post-treatment rather than after a longer period. Further research is still needed to fully understand the specific pathways and mechanisms involved and to determine the influence of rTMS on sleep disorders in patients with PD over the long term.

## 5. Conclusions

Ten sessions of 20 Hz rTMS over both parietal cortexes possibly improve sleep quality as measured both subjectively and objectively in patients with PD. Secondarily, these improvements are parallel to the improvements in motor and mood dysfunction.

## Figures and Tables

**Table 1:** Demographic and clinical rating scales of the studied groups.

Variables	Sham Group (*n* = 8)	Active Group (*n* = 16)	Z or x^2^	*p*-Value
Age (mean ± SD) Median (IQR)	60.21 ± 1.64 59(3)	61.82 ± 3.48 62(5)	−1.927	0.053
Gender				
Males, number (%)	6(75%)	7(43.8%)	2.098	0.211
Females	2(25%)	9(56.3%)		
Duration of illness (mean ± SD) Median (IQR)	5.87 ± 4.08 4.5(6.5)	7.12 ± 3.48 9(6.5)	−0.774	0.439
BMI (kg/m^2^) Median (IQR)	25.69 ± 3.57 26.15(6.18)	26.95 ± 5.2 26.15(6.74)	-	-
MDS-UPDRS (motor section parts II and III) MDS-UPDRS (mean ± SD)	60.37 ± 33.41	66.31 ± 23.41	−0.532	0.601
Beck Depression Rating Scale (HDRS) (mean ± SD)	15.42 ± 6.57	14.87 ± 6.35	−0.034	0.973
Parkinson’s Disease Sleep Scale (PDSS) (mean ± SD)	14.14 ± 2.19	14.12 ± 2.55	−0.031	0.975

**Table 2:** The mean value (pre- and 10 days post-rTMS sessions) of the different rating scales among the studied groups.

	Baseline Assessment	Follow-Up 10 Days after Sessions [% of Improvement]	Wilcoxon Measure Analysis	Effect Size	Friedman Measure Analysis Time × Group Interaction
Parkinson’s Disease Sleep Scale (PDSS)					
Sham group (*n* = 8) Median (IQR)	14.14 ± 2.19 14(4)	13.14 ± 3.80 14(4), [1(12.5%)] #	Z = −1.000, *p* = 0.317	[0.756] ^a^	*p* = 0.001 *
Real group (*n* = 16) Median (IQR)	14.12 ± 2.55 14(3)	7.62 ± 3.89 6.5(3.75), [13(81.25%)] #	Z = −3.524, *p* = 0.001 *	[3.724] ^a^	
Mann–Whitney at each time of assessment	Z = −0.031, *p* = 0.975	Z = −2.818, *p* = 0.0005 *			
MDS-UPDRS (motor sections II and III)					
Sham group (*n* = 8) Median (IQR)	60.37 ± 33.41 58.5(70)	56.75 ± 37.64 57(72), [1(12.5%)] #	Z = −1.604, *p* = 0.109	[1.377] ^a^	*p* = 0.001 *
Real group (*n* = 16) Median (IQR)	66.31 ± 23.41 63.5(42.75)	45.25 ± 20.86 38.5(27.25), [9(56.25%)] #	Z = −3.521, *p* = 0.001 *	[3.71] ^a^	
Mann–Whitney at each time of assessment	Z = −0.523, *p* = 0.601	Z = −0.769, *p* = 0.442			
Beck Depression Inventory Scale (BDI)					
Sham group (*n* = 8) Median (IQR)	15.42 ± 6.57 16(9)	15.28 ± 6.67 16(9), [0(0%)] #	Z = −1.000, *p* = 0.317	[0.756] ^a^	*p* = 0.001 *
Real group (*n* = 16) Median (IQR)	14.87 ± 6.35 15.5(10.5)	8.87 ± 5.85 9(9.75), [12(75%)] #	Z = −3.412, *p* = 0.001 *	[3.269] ^a^	
Mann–Whitney at each time of assessment	Z = −0.034, *p* = 0.973	Z = −1.848, *p* = 0.065			

^a^ Cohen’s d coefficient, IQR; interquartile range, # the percentage of patients who developed improvement >= 30%, * significant *p* value.

**Table 3:** The mean value (pre- and post-rTMS sessions) of polysomnography parameters (part 1) among the studied groups.

	Baseline Assessment	Follow Up 10 Days after the Sessions [% of Improvement]	Wilcoxon Measure Analysis	Effect Size	Friedman Measure Analysis Time × Group Interaction
Sleep Latency (min) [normal sleep latency = normal: <20 min]					
Sham group (*n* = 8) Median (IQR)	29.25 ± 7.92 28.5(17)	29.12 ± 8.40 28.5(16.5)	Z = −0.106, *p* = 0.916	[0.075] ^a^	*p* = 0.003 *
Real group (*n* = 16) Median (IQR)	28.87 ± 17.47 25(29.25)	22.68 ± 15.17 18(21.25)	Z = −3.427, *p* = 0.001 *	[3.322] ^a^	
Mann–Whitney at each time of assessment	Z = −0.400, *p* = 0.689	Z = −1.594, *p* = 0.111			
Rapid eye movement (REM) latency (min) [REM latency = ≥90 min]					
Sham group (*n* = 8) Median (IQR)	42.39 ± 20.04 45.07(42)	41.25 ± 21.06 43.5(44)	Z = −1.89, *p* = 0.059	[1.796] ^a^	*p* = 0.007 *
Real group (*n* = 16)	35.75 ± 21.41 30(40.5)	51.50 ± 23.76 52(47.75)	Z = 3.519, *p* = 0.001 *	[3.701] ^a^	
Mann–Whitney at each time of assessment	Z = −0.921, *p* = 0.357	Z = −1.136, *p* = 0.256			
Number of awakenings [normal > five (more than five indicates sleep fragmentation]					
Sham group (*n* = 8) Median (IQR)	5.62 ± 1.40 5.5(2.5)	6 ± 1.21 5.5(2)	Z = 0.966, *p* = 0.334	[0.727] ^a^	*p* = 0.157
Real group (*n* = 16) Median (IQR)	4.93 ± 3.24 4(3.5)	3.43 ± 2.15 3.5(4)	Z = −2.386, *p* = 0.017 *	[1.486] ^a^	
Mann–Whitney at each time of assessment	Z = −1.399, *p* = 0.162	Z = −2.706, *p* = 0.007 *			
Wake-after-sleep onset index (WASO) [normal WASO = <20–30 min					
Sham group (*n* = 8) Median (IQR)	33.88 ± 8.83 35.5(15)	36.37 ± 8.26 38(12.75)	Z = −0.677, *p* = 0.498	[0.493] ^a^	*p* = 0.001 *
Real group (*n* = 16) Median (IQR)	39.5 ± 14.19 40(15)	22.93 ± 11.79 23(17.25)	Z = −3.442, *p* = 0.001 *	[3.378] ^a^	
Mann–Whitney at each time of assessment	Z = −1.137, *p* = 0.255	Z = −0.2.545, *p* = 0.011			
Wake % TST [Total Wake Time/Total Sleep Time) × 100 = <10%]					
Sham group (*n* = 8) Median (IQR)	6.85 ± 5.10 6.9(10.4)	6.53 ± 4.72 6.9(9.4)	Z = −1.46, *p* = 0.144	[1.205] ^a^	*p* = 0.007 *
Real group (*n* = 16) Median (IQR)	7 ± 4.49 6.7(9.53)	3.5 ± 3.57 1.5(7.45)	Z = −2.77, *p* = 0.006 *	[1.92] ^a^	
Mann–Whitney at each time of assessment	Z = −0.123, *p* = 0.902	Z = −1.84, *p* = 0.065			
N1 % TST [(normal stage 1 sleep = 5%] non-REM sleep					
Sham group (*n* = 8) Median (IQR)	1.51 ± 0.72 1.6(0.6)	1.11 ± 0.75 1.25(1.5)	Z = −2.207, *p* = 0.027 *	[2.495] ^a^	*p* = 0.001 *
Real group (*n* = 16) Median (IQR)	2.56 ± 1.60 2.15(2.2)	1.51 ± 1.11 1.15(1.4)	Z = −3.18, *p* = 0.001 *	[2.621] ^a^	
Mann–Whitney at each time of assessment	Z = −1.933, *p* = 0.053	Z = −0.553, *p* = 0.58			
N2%TST [normal stage 2 sleep = 50%] non-REM sleep					
Sham group (*n* = 8) Median (IQR)	67.78 ± 6.89 66.65(11.72)	66.77 ± 5.63 65(9.35)	Z = −1.524, *p* = 0.128 *	[1.279] ^a^	*p* = 0.088
Real group (*n* = 16) Median (IQR)	64.08 ± 10.67 65.05(14.72)	60.10 ± 9.44 62.25(13.75)	Z = −2.13, *p* = 0.033 *	[1.258] ^a^	
Mann–Whitney at each time of assessment	Z = −0.796, *p* = 0.426	Z = −1.871, *p* = 0.061			
N3%TST [stage N3 sleep is considered “deep sleep” or slow wave sleep normal = 20%] non-REM sleep					
Sham group (*n* = 8) Median (IQR)	15.97 ± 6.40 15.9(6.13)	15.7 ± 6.55 15(7.82)	Z = −0.631, *p* = 0.528	[0.458] ^a^	*p* = 0.827
Real group (*n* = 16) Median (IQR)	18.49 ± 10.46 18.7(16.58)	18.62 ± 9.66 17.5(15.5)	Z = −0.085, *p* = 0.932	[0.043] ^a^	
Mann–Whitney at each time of assessment	Z = −0.368, *p* = 0.713	Z = −0.705, *p* = 0.481			
REM %TST [REM %TST = normal 25%]					
Sham group (*n* = 8) Median (IQR)	18.36 ± 8.1 17.5(11.5)	17.12 ± 8.44 15.5(15.5)	Z = −1.43, *p* = 0.15	[1.172] ^a^	*p* = 0.088
Real group (*n* = 16) Median (IQR)	14.66 ± 6.78 16.6(9.78)	16.68 ± 6.72 18.5(8.25)	Z = −2.72, *p* = 0.006 *	[1.855] ^a^	
Mann–Whitney at each time of assessment	Z = −0.857, *p* = 0.391	Z = −0.154, *p* = 0.878			

^a^ Cohen’s d coefficient; * significant *p* value; TST; total sleep time, IQR; interquartile range, HR; heart rate, N1; stage 1 (light sleep), N2; stage 2, N3; stage 3 (deep sleep), REM; rapid eye movement sleep, WASO; wake-after-sleep onset index. WASO is usually expressed as the total duration of wakefulness (in minutes) during the sleep period. A low percentage of stage N2 sleep may be a result of sleep fragmentation with increased REM (N3). Data in brackets indicate data from our laboratory.

**Table 4:** The mean value (pre- and post-rTMS sessions) of polysomnography parameters (part II) among the studied groups.

	Baseline Assessment	Follow Up 10 Days after Sessions [% of Improvement]	Wilcoxon Measure Analysis	Effect Size	Friedman Measure Analysis Time × Group Interaction
Desaturation index [normal ODI < 5 events/hour]					
Sham group (*n* = 8) Median (IQR)	4.94 ± 3.58 5.7(7.3)	5.4 ± 3.94 5.7(9.4)	Z = −1.34, *p* = 0.18	[1.076] ^a^	*p* = 0.225
Real group (*n* = 16) Median (IQR)	6.67 ± 4.84 5.9(9.1)	4.89 ± 3.12 5.7(5.57)	Z = −1.534, *p* = 0.125	[0.83] ^a^	
Mann–Whitney at each time of assessment	Z = −0.637, *p* = 0.524	Z = −0.062, *p* = 0.951			
Average oxygen saturation SpO_2_					
Sham group (*n* = 8) Median (IQR)	93.43 ± 1.81 94(3)	91.85 ± 3.33 92(3)	Z = −1, *p* = 0.317	[0.756] ^a^	*p* = 0.405
Real group (*n* = 16) Median (IQR)	92.93 ± 2.58 94(2)	94.21 ± 2.11 94.5(3.5)	Z = −0.998, *p* = 0.318	[0.515] ^a^	
Mann–Whitney at each time of assessment	Z = −0.032, *p* = 0.975	Z = −1.802, *p* = 0.071			
Arousal index (/h) (normal: <five events/hour)					
Sham group (*n* = 8) Median (IQR)	12.74 ± 14.24 5(16.7)	12.23 ± 12.5 6(13)	Z = −0.105, *p* = 0.917	[0.074] ^a^	*p* = 0.033 *
Real group (*n* = 16) Median (IQR)	9.95 ± 7.11 9.9(10.63)	8.11 ± 6.51 6.7(11)	Z = −2.94, *p* = 0.003 *	[2.168] ^a^	
Mann–Whitney at each time of assessment	Z = −0.429, *p* = 0.668	Z = −0.460, *p* = 0.645			
Periodic leg movement index (PLM index) (/h) [normal = PLM index = <five events/hour]					
Sham group (*n* = 8) Median (IQR)	8.42 ± 8.34 4.38(14.95)	7.87 ± 8.14 3(16)	Z = −1.214, *p* = 0.225	[0.95] ^a^	*p* = 0.001 *
Real group (*n* = 16) Median (IQR)	6.75 ± 6.43 4.39(9.5)	2.08 ± 3.09 1.56(2.26)	Z = −2.97, *p* = 0.003*	[2.217] ^a^	
Mann–Whitney at each time of assessment	Z = −0.553, *p* = 0.580	Z = −2.112, *p* = 0.035 *			
Apnea, hypopnea index per hour AHI/h [normal: AHI < five events/hour]					
Sham group (*n* = 8) Median (IQR)	8.24 ± 4.34 5.13(7.4)	9.23 ± 5.63 5.13(11.23)	Z = −1.604, *p* = 0.109	[1.377] ^a^	*p* = 0.134
Real group (*n* = 16) Median (IQR)	8.09 ± 5.36 8.84(9.8)	4.01 ± 1.95 4.77(1.8)	Z = −2.553, *p* = 0.011 *	[1.658] ^a^	
Mann–Whitney at each time of assessment	Z = −0.226, *p* = 0.821	Z = −2.34, *p* = 0.019 *			

^a^ Cohen’s d coefficient, * significant *p* value, IQR; interquartile range.

**Table 5:** The correlation between the mean changes in different rating scales (pre- and post-rTMS sessions) and the mean difference (pre-post sessions) of the parameters of polysomnography among studied groups.

Variables		PDSS	UPDRS Part III	Beck Depression Rating Scale (HDRS)
Number of awakenings	r	0.272	0.272	−0.054
	*p*-value	0.198	0.198	0.801
Wake-after-sleep onset index	r	0.355	0.605	−0.421
	*p*-value	0.088	0.002 *	0.041 *
Wake % TST	r	0.371	0.406	−0.148
	*p*-value	0.074	0.049 *	0.490
Sleep latency (min)	r	0.466 *	0.639	−0.333
	*p*-value	0.022 *	0.001 *	0.111
REM latency (min)	r	−0.764	−0.706	0.221
	*p*-value	<0.0001 *	<0.0001 *	0.299
N1 % TST (stage 1 non-REM)	r	0.377	0.552	−0.192
	*p*-value	0.070	0.005 *	0.368
N2 % TST (stage 2 non-REM)	r	0.006	0.079	0.105
	*p*-value	0.978	0.713	0.624
N3 % TST (stage 3 deep sleep)	r	−0.101	−0.136	0.080
	*p*-value	0.638	0.527	0.712
REM % TST	r	−0.333	−0.682	0.560
	*p*-value	0.112	<0.0001 *	0.004 *
PLMs index (/h)	r	0.276	0.274	−0.083
	*p*-value	0.192	0.195	0.701
Arousal index (/h)	r	0.159	0.261	−0.357
	*p*-value	0.457	0.217	0.087
AHI/h	r	0.261	0.488	−0.207
	*p*-value	0.230	0.018 *	0.342

* significant *p* value.

## Data Availability

The original contributions presented in this study are included in this article. Further inquiries can be directed to the corresponding author.
